# Suppression of mitochondrial respiration by hydrogen sulfide in hibernating 13-lined ground squirrels

**DOI:** 10.1016/j.freeradbiomed.2021.04.009

**Published:** 2021-04-20

**Authors:** Birgitte S. Jensen, Sibile Pardue, Brynne Duffy, Christopher G. Kevil, James F. Staples, Angela Fago

**Affiliations:** a Department of Biology, Aarhus University, Aarhus C, 8000, Denmark; b Department of Biology, University of Western Ontario, London, ON N6A 5B8, Canada; c Department of Pathology, Louisiana State University Health Sciences Center, Shreveport, LA, 71130, USA

**Keywords:** H_2_S, Hypometabolism, *Ictidomys tridecemlineatus*, sulfide:quinone oxidoreductase, Torpor

## Abstract

Hibernating mammals may suppress their basal metabolic rate during torpor by up to 95% to reduce energy expenditure during winter, but the underlying mechanisms remain poorly understood. Here we show that hydrogen sulfide (H_2_S), a ubiquitous signaling molecule, is a powerful inhibitor of respiration of liver mitochondria isolated from torpid 13-lined ground squirrels, but has a weak effect on mitochondria isolated during summer and hibernation arousals, where metabolic rate is normal. Consistent with these *in vitro* effects, we find strong seasonal variations of *in vivo* levels of H_2_S in plasma and increases of H_2_S levels in the liver of squirrels during torpor compared to levels during arousal and summer. The *in vivo* changes of liver H_2_S levels correspond with low activity of the mitochondrial H_2_S oxidizing enzyme sulfide:quinone oxidoreductase (SQR) during torpor. Taken together, these results suggest that during torpor, H_2_S accumulates in the liver due to a low SQR activity and contributes to inhibition of mitochondrial respiration, while during arousals and summer these effects are reversed, H_2_S is degraded by active SQR and mitochondrial respiration rates increase. This study provides novel insights into mechanisms underlying mammalian hibernation, pointing to SQR as a key enzyme involved in the control of mitochondrial function.

## 1. Introduction

Hibernation is a powerful way of suppressing metabolic rate which allows some mammalian species to save energy during winter [1]. Hibernating 13-lined ground squirrels (*Ictidomys tridecemlineatus*) spend most of the winter in torpor, a state where metabolic rate is suppressed by ~95% and body temperature (T_b_) drops to ~5 °C. Torpor bouts are regularly interrupted by short arousals of ~10–12 h to interbout euthermia (IBE), during which metabolic rate and T_b_ rapidly return to normal levels [2]. The mechanisms underlying the dramatic metabolic suppression of torpor are complex and not fully understood [3]. It is, however, well established that mitochondrial function parallels the whole-animal metabolic variation between torpor and IBE during winter hibernation. As case in point, respiration rates under phosphorylating conditions of liver mitochondria isolated from 13-lined ground squirrels are ~70% lower in torpor than in IBE [4,5]. This suppression of mitochondrial metabolism occurs without major changes in either tissue mitochondrial density [6] or mitochondrial content of electron transport system (ETS) complexes [7]. Instead, changes in the activities of ETS complex I and II correspond with changes in the phosphorylation state of their subunits [8]. While these post-translational modifications are important in the switch of mitochondrial function between torpor and IBE during winter hibernation, it is possible that additional control mechanisms in the ETS can further contribute to suppress respiration rates.

The gasotransmitter hydrogen sulfide (H_2_S) has been implicated in the metabolic depression of hibernation due to its potent *in vitro* inhibitory effect on cytochrome *c* oxidase [9-11], the O_2_ consuming complex IV of the ETS. In support of this view, studies have reported that exogenous H_2_S is capable of inducing a state of suspended animation in mice [12] similar to natural hibernation, although some controversy exists [3]. Moreover, other studies by us and others have revealed significant changes in the endogenous levels and composition of sulfur metabolites in the plasma of 13-lined squirrels [13], in the blood of brown bears [14] and in the lung of Syrian hamsters [15] during torpor or hibernation. As a signaling molecule, H_2_S is ubiquitously produced by several enzymes [16] and it has been reported that H_2_S synthesis increases during torpor by increased activity of cystathionine β-synthase (CBS) and increased availability of substrates cysteine and cystathionine [3,13,15]. H_2_S is also continuously oxidized in the mitochondria by the membrane-bound sulfide:quinone oxidoreductase (SQR), controlling H_2_S homeostasis and supplying electrons to complex III of the ETS [17]. Here, we present evidence supporting that in 13-lined ground squirrels SQR activity is inhibited during torpor, which leads to accumulation of H_2_S with consequent inhibition of mitochondrial respiration. Taken together, these results demonstrate that H_2_S plays a key role in the metabolic suppression of mammalian hibernation.

## 2. Materials and methods

### 2.1. Animals

13-lined ground squirrels (*Ictidomys tridecemlineatus*) were trapped during late spring in Carman, MB, Canada (49°30′N, 98°01′W) with permission from Manitoba Conservation or bred in captivity at University of Western Ontario following established protocols [18]. During summer, body temperature radio telemeters (TA-ETAF10, Data Sciences International, St. Paul, MN) were implanted in the abdomen of animals [5]. In October, the animals were transferred to a hibernation chamber where the temperature was reduced by 1 °C/day until it reached 5 °C, at which point it was held constant for the duration of the hibernation season (January–March) and the photoperiod was set to 2 h light: 22 h dark [7].

Animals were euthanized and tissues sampled during three stages: 1) *summer;* euthermic animals, and winter hibernating, including 2) *torpor;* defined by a stable T_b_ of 5° ± 3 °C for 4–5 days, and 3) *IBE*; defined by a stable T_b_ near 37 °C for 2–3 h following a spontaneous arousal. Summer and IBE animals were euthanized by a lethal dose of Euthanyl (54 mg/100g), which does not affect mitochondrial metabolism [19]. Torpid animals were euthanized by cervical dislocation, as handling and anesthetic injection would initiate arousal.

All animal procedures were approved by the local Animal Care Committee (protocol 2012-016) of the University of Western Ontario and conformed to the guidelines of the Canadian Council on Animal Care.

### 2.2. Plasma and liver sampling

Blood was collected in heparin-coated vacutainers from the inferior vena cava in summer, and in winter, blood was collected following decapitation, as venous blood pressure was insufficient. Blood samples were centrifuged and 250 μL plasma was added to 800 μL conservation buffer (100 mM Tris-HCl, pH 9.5, 0.1 mM diethylenetriaminepenta-acetic acid, DTPA), where the alkaline pH converts H_2_S to the anionic form HS^−^ avoiding dissipation of H_2_S. Likewise, ~200 mg liver was preserved in 800 μL conservation buffer. All samples were snap-frozen in liquid N_2_ for later measurement of bioavailable H_2_S. The remaining liver was stored in ice-cold homogenization buffer (250 mM sucrose, 10 mM HEPES, 1 mM EGTA, pH 7.4) and used immediately for isolation of mitochondria.

### 2.3. Bioavailable H_2_S

Bioavailable H_2_S was measured using a fluorescent monobromobimane assay as previously described [20], following extensively validated and refined protocols [21,22]. Sample H_2_S was derivatized by 30 min incubation with excess monobromobimane at 1% O_2_ using a hypoxic chamber in the dark at room temperature. The product of the reaction of monobromobimane with H_2_S, sulfide dibimane, was separated from unreacted monobromobimane by RP-HPLC using a fluorescent detector (λ_ex_: 390 nm and λ_em_: 475 nm) and quantified after calibration with known H_2_S concentrations. Although monobromobimane does not significantly extrude sulfur of proteins [23], some protein-bound sulfane sulfur mobilization may occur under certain conditions [24].

### 2.4. Isolation of mitochondria

We used liver mitochondria, in which metabolic suppression is particularly pronounced during torpor compared with other tissues [5,25,26]. Mitochondria were isolated using differential centrifugation and purified by a Percoll gradient [27]. Purified mitochondria were suspended in 1 ml of homogenization buffer with 1% (w/v) BSA, and used immediately for measurement of mitochondrial respiration and H_2_S consumption.

### 2.5. Mitochondrial respiration

Oxygen consumption of isolated liver mitochondria was measured using an Oxygraph-2K high-resolution respirometer (Oroboros Instruments, Austria) as previously described [7]. State 2 respiration rates were determined after the addition of rotenone (2.5 μM, dissolved in 95% ethanol) and succinate (6 mM, dissolved in respiration medium) since the suppression of liver mitochondrial metabolism during torpor is greatest with succinate as oxidative substrate [4,5]. When the concentration of O_2_ within the respirometry chambers reached ~160 μM, various concentrations of Na_2_S were added (1.6–3.9 μM), to test the effect of H_2_S on respiration. Anaerobic Na_2_S stock solutions were freshly prepared (Na_2_S•9H_2_O in N_2_-equilibrated milli-Q water, ~8 mM).

### 2.6. Mitochondrial H_2_S consumption

An amperometric H_2_S-specific microsensor (customized SULF-microsensor, Unisense A/S, Aarhus, Denmark) coupled to a Unisense PA2000 amplifier was used to determine mitochondrial consumption rates of externally added H_2_S (as Na_2_S). The microsensor tip is covered by a membrane that only allows H_2_S to selectively pass and react with the electrolyte solution. To identify the site of H_2_S consumption, summer mitochondria were pre-incubated for 10 min with rotenone (2.5 μM) or antimycin A (2.5 μM) to inhibit ETS complex I and III, respectively, before H_2_S consumption was measured. Mitochondrial H_2_S consumption was also measured in sonicated mitochondria (2 × 10 s on ice).

### 2.7. SQR activity assay

SQR activity of previously frozen liver mitochondria from summer, torpid and IBE squirrels was obtained by following the reduction rate of decyl-ubiquinone at 275 nm upon Na_2_S addition [28].

### 2.8. Statistical analysis

Statistical analyses were performed using Prism (GraphPad Software Inc, La Jolla, CA, USA). A Shapiro-Wilk test confirmed normal distribution of data. Significant differences among hibernation states were assessed by one-way ANOVA with Tukey’s multiple comparisons test. Significant differences are indicated (*P < 0.05). Data are reported as means ± SEM, unless otherwise stated.

## 3. Results and discussion

**Fig. 1.**
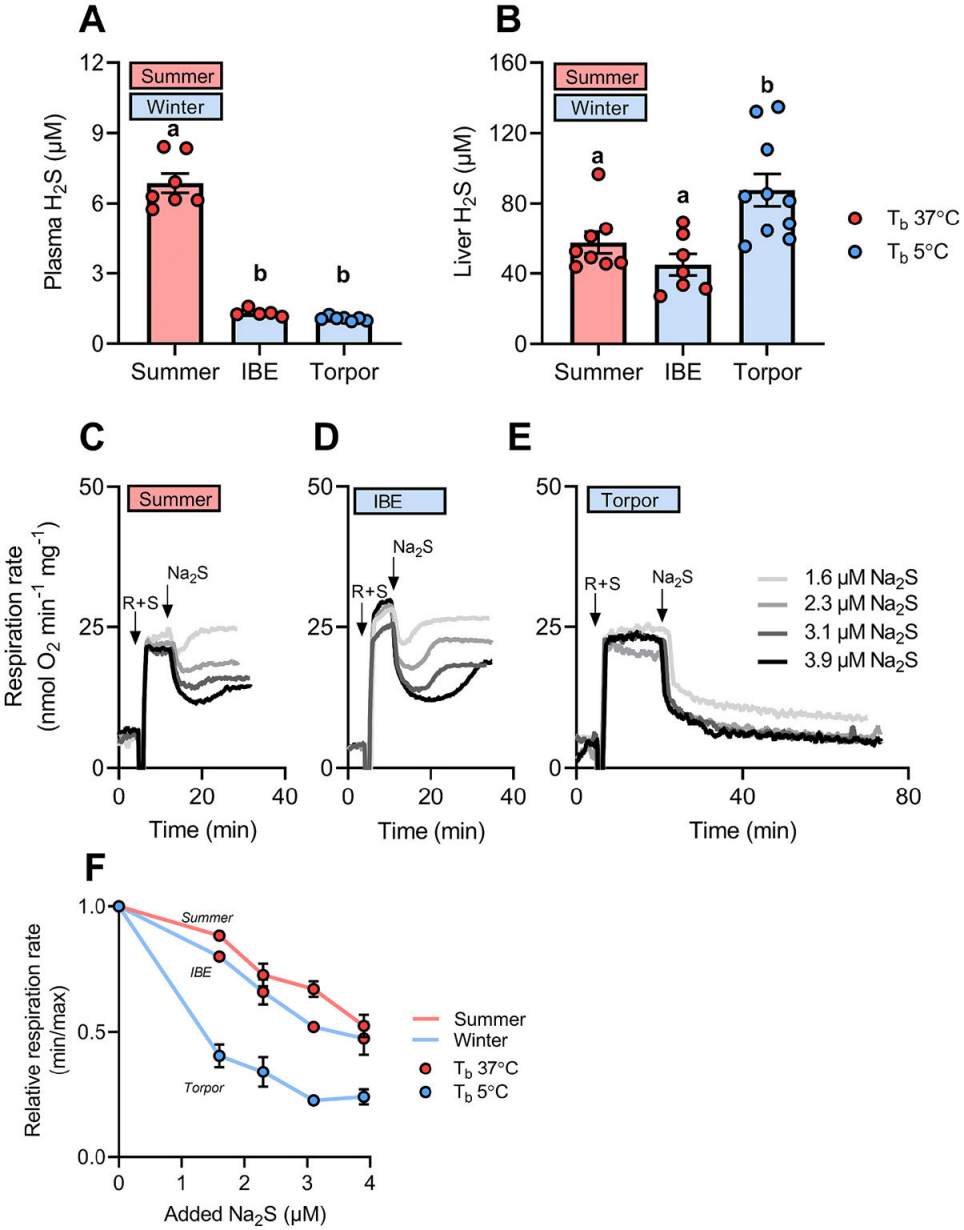
In vivo *H_2_S levels and H_2_S-induced inhibition of mitochondrial respiration.* (A and B) H_2_S levels in plasma and liver of summer and winter hibernating squirrels (interbout euthermia [IBE] and torpor). Individual values are shown with mean ± SEM. Different letters (a–b) indicate a significant difference, P < 0.05. (C–E) Representative traces of respiration rate of liver mitochondria isolated during summer, IBE and torpor. Arrows indicate additions of 2.5 μM rotenone (R), 6 mM succinate (S) and H_2_S (added as Na_2_S). Mitochondria (10–30 μl, ~200 μg protein) were transferred to the respiration chamber containing 2 ml of mitochondrial respiration medium (0.5 mM EGTA, 3 mM MgCl_2_, 60 mM l-lactobionate, 20 mM taurine, 10 mM KH_2_PO_4_, 20 mM HEPES, 110 mM sucrose, 1 g/l fatty acid-free BSA, pH 7.1) at 37 °C and constant stirring (750 rpm). Electrodes were calibrated daily to 0% oxygen (using a yeast suspension) and air-saturation. Respiration rates were expressed relative to protein concentration determined using a protein assay dye (Bio-Rad). (F) Relative respiration rate (minimum rate after Na_2_S addition normalized to initial respiration rate) plotted as a function of added Na_2_S. Data are shown as mean ± SD, N = 7 (summer) and N = 2 (IBE and torpor).

We first measured bioavailable H_2_S in plasma and liver from winter (torpid and IBE) and summer active 13-lined squirrels to search for *in vivo* changes. Plasma H_2_S levels were significantly higher in summer than in winter hibernating squirrels, where levels did not differ significantly between torpor and IBE (Fig. 1A). H_2_S in plasma reflects extracellular enzymatic production by cystathionine γ-lyase (CSE) and CBS secreted by endothelial cells and liver [29] or H_2_S produced in the red blood cells [14,30]. Interestingly, similar plasma H_2_S values were detected in winter hibernating and summer brown bears using the same method [14], suggesting common seasonal patterns of sulfide transport in the circulation of hibernators.

In the liver of 13-lined squirrels, bioavailable H_2_S was significantly higher in torpor compared with IBE and summer (Fig. 1B). To determine the effect of H_2_S on liver mitochondria during torpor, we measured H_2_S-dependent respiration rates in isolated mitochondria using high-resolution respirometry. In all three states, H_2_S additions rapidly inhibited respiration rates (Fig. 1C-E) in a concentration-dependent fashion (Fig. 1F), but the time course and magnitude differed. In IBE and summer mitochondria, the H_2_S-dependent inhibition of respiration was modest and transient, and respiration rates largely recovered after few minutes (Fig. 1C and D), similar to what has been observed in human cells [31]. Conversely, in mitochondria isolated from torpid squirrels, inhibition of respiration was more pronounced and was maintained for at least 70 min at all H_2_S concentrations examined (Fig. 1E and F). Although these effects can in principle originate from different sensitivities of complex IV to H_2_S, a previous study found no difference in *in vitro* complex IV activity measured in liver mitochondria from 13-lined squirrels in torpor and IBE [7]. Taken together, these data suggest that the strong inhibition of complex IV by H_2_S observed during torpor (Fig. 1E) is achieved not by post-translational modifications of complex IV, but by changes in the mitochondrial rate of H_2_S oxidation, which would alter the levels of free H_2_S available for inhibition. Since free H_2_S in tissues is readily lost during isolation of mitochondria, these effects on complex IV function have remained unnoticed in previous *in vitro* studies.

**Fig. 2.**
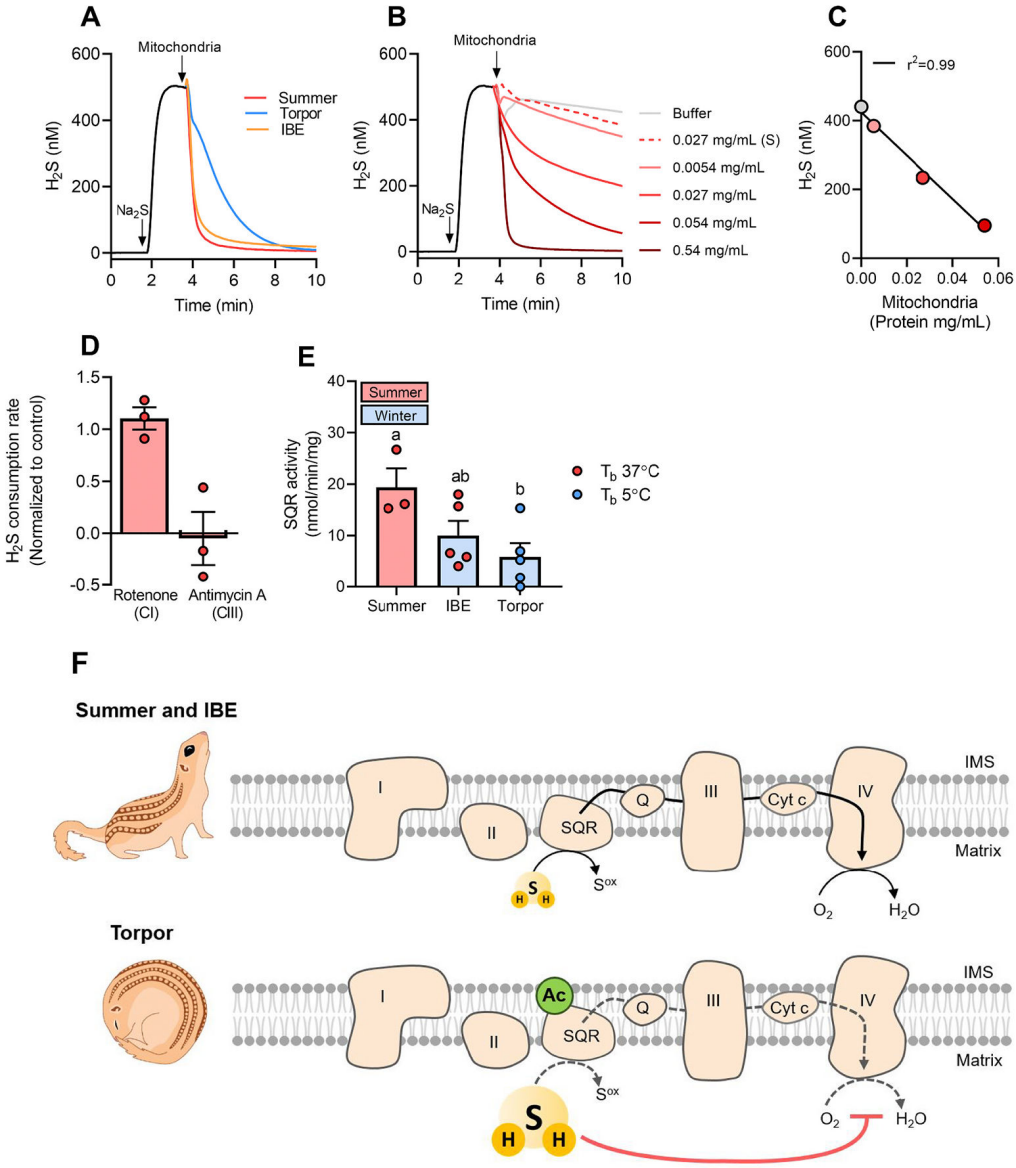
Decreased mitochondrial H*_2_S oxidation and SQR activity during torpor.* (A) Representative traces of H_2_S consumption rates by liver mitochondria (0.5 mg protein/mL) freshly isolated from a summer active, an IBE and a torpid squirrel shown as mean of technical duplicates. Consumption rate was measured using a H_2_S microsensor introduced through a thin capillary into a sealed 1 ml glass chamber containing PBS buffer (1.37 mM NaCl, 28.8 μM KCl, 100 μM Na_2_HPO_4_, 17.6 μM KH_2_PO_4_ pH 7.4) at constant stirring and submerged in a water bath at 37 °C. Na_2_S (500 nM) was injected in the chamber via a thin injection port, followed by injections of freshly isolated liver mitochondria from torpid, IBE and summer animals, and the H_2_S consumption was followed for ~20 min. The microsensor was calibrated with freshly made anaerobic Na_2_S stock solutions. (B) H_2_S consumption by summer mitochondria at various amounts of mitochondria. Controls with buffer (grey) and sonicated (S) mitochondria (0.027 mg protein/mL, dashed red line) are shown. (C) H_2_S concentrations at 8 min in (B) plotted as a function of mitochondrial protein concentration. (D) H_2_S consumption rate of liver mitochondria from summer squirrels in the presence of rotenone or antimycin A relative to control. Individual values are shown with mean ± SEM. (E) SQR activity of liver mitochondria. Individual values are shown with mean ± SEM. Different letters indicate a significant difference, P < 0.05. Frozen mitochondria were carefully thawed and suspended in 20 mM Tris-HCl buffer, pH 7.8 in sealed 1-cm-quartz cuvettes containing KCN (2 mM), rotenone (40 μM) and decyl-ubiquinone (150 μM). The background rate was followed for ~5 min at 275 nm, 37 °C, before the reaction was initiated with 100 μM Na_2_S and followed for 5–10 min in technical triplicates. The SQR activity was calculated as the background-corrected ΔA_275_/time over ~2 min intervals, using an extinction coefficient of 15 L^−^mmol^−^ cm^−1 28^ and normalized to the protein concentration, calculated from the initial A_280_ using an experimentally determined extinction coefficient calculated from a standard curve of known mitochondrial protein concentration. (F) Proposed H_2_S-dependent control of mitochondrial respiration in 13-lined ground squirrels during summer and IBE (upper panel) and torpor (lower panel). Ac: Putative acetylation, Cyt *c*: cytochrome *c*, Q: ubiquinone, IMS: intermembrane space, S^ox^: Oxidized sulfur, e.g. thiosulfate or sulfite.

To establish whether the H_2_S oxidation capacity of mitochondria was lower during torpor, we examined whether and how liver mitochondria isolated from torpid, IBE and summer squirrels differed in the oxidation rate of externally added H_2_S using an amperometric H_2_S-sensitive microsensor. We found that torpid mitochondria oxidized H_2_S slower than IBE and summer mitochondria (Fig. 2A) and that the rate of H_2_S oxidation was linearly dependent on the amount of mitochondria added (Fig. 2B and C). In addition, disruption of mitochondria membrane by sonication (Fig. 2B and C) or addition of antimycin A, an inhibitor of complex III, to intact mitochondria (Fig. 2D) abolished H_2_S oxidation, whereas rotenone, an inhibitor of complex I, had no effect (Fig. 2D). Taken together, these data indicate that the membrane enzyme SQR, which is positioned downstream of complex I and upstream of complex III, was responsible for H_2_S oxidation. To confirm these results, we measured SQR activity in liver mitochondria and found that it was significantly lower during torpor compared with summer, whereas activity during IBE was intermediate (Fig. 2E). In agreement with these results, SQR knockdown in human cancer cells increases the inhibitory effect of H_2_S on respiration [32]. The molecular mechanism underlying suppression of SQR activity during torpor in hibernating squirrels remains to be identified. While changes in SQR expression during IBE and torpor may be one way of changing SQR activity, the suppression of liver mitochondrial respiration happens very rapidly during entrance into torpor [33]. Moreover, the low temperatures during torpor and early arousal cause a near complete suppression of transcription and translation [34], whereby upregulation of SQR during arousal appears unlikely. Rapid and reversible posttranslational modifications may be offer a more timely and energetically favorable mechanism for changing SQR activity. Specifically, protein acetylation appears as a pervasive modification during torpor in 13-lined squirrel liver [34] and an unknown mitochondrial protein with the same molecular mass as SQR (~50 kDa) was found to be differentially acetylated between torpor and IBE in 13-lined ground squirrel liver mitochondria [8], consistent with the presence in mouse SQR of a acetylation site [35]. Future targeted transcriptomic and proteomic studies are needed to unravel SQR regulation in hibernating 13-lined squirrels. The H_2_S-sensitive transcription factor nuclear factor-erythroid 2-related factor 2 (Nrf2), which increases during torpor in the liver 13-lined squirrels [36], may also be involved in regulating SQR expression during hibernation and especially between summer and winter, as suggested in mice fibroblast [37]. This aspect too remains to be investigated.

Based on our data, we propose a model that accounts for the H_2_S-mediated depression of mitochondrial respiration during hibernation (Fig. 2F). During summer and IBE, H_2_S is degraded by active SQR and mitochondrial respiration is normal. During torpor, SQR activity is inhibited, which leads to a lower rate of H_2_S oxidation and to a sufficient build-up of H_2_S to inhibit complex IV. The consequent depression of mitochondrial respiration during torpor would then match oxygen consumption with the low oxygen supply deriving from low rates of pulmonary ventilation and heart rates [1]. In conclusion, we found a powerful inhibitory effect of H_2_S on mitochondrial respiration of hibernating 13-lined ground squirrels during torpor, paralleled by increased *in vivo* levels of bioavailable H_2_S in the liver. Although the proposed mechanism does not exclude a possible role of H_2_S-synthetising enzymes, the strongly depressed oxidation of H_2_S by the mitochondrial enzyme SQR provides compelling evidence that the H_2_S catabolic pathway is a key regulatory site for the regulation of metabolism during hibernation.

## Abbreviations:

CBS: Cystathionine β-synthase
CSE: Cystathionine γ-lyase
ETS: Electron transport system
H_2_S: Hydrogen sulfide
IBE: Interbout euthermia
SQR: sulfide:quinone oxidoreductase
T_b_: Body temperature.
